# Three-Dimensional Characterization of the Vascular Bed in Bone Metastasis of the Rat by Microcomputed Tomography (MicroCT)

**DOI:** 10.1371/journal.pone.0017336

**Published:** 2011-03-28

**Authors:** Hervé Nyangoga, Philippe Mercier, Hélène Libouban, Michel Félix Baslé, Daniel Chappard

**Affiliations:** INSERM, U922 – LHEA, IRIS-IBS Institut de Biologie en Santé, CHU d'Angers, Angers, France; Ohio State University, United States of America

## Abstract

**Background:**

Angiogenesis contributes to proliferation and metastatic dissemination of cancer cells. Anatomy of blood vessels in tumors has been characterized with 2D techniques (histology or angiography). They are not fully representative of the trajectories of vessels throughout the tissues and are not adapted to analyze changes occurring inside the bone marrow cavities.

**Methodology/Principal Findings:**

We have characterized the vasculature of bone metastases in 3D at different times of evolution of the disease. Metastases were induced in the femur of Wistar rats by a local injection of Walker 256/B cells. Microfil®, (a silicone-based polymer) was injected at euthanasia in the aorta 12, 19 and 26 days after injection of tumor cells. Undecalcified bones (containing the radio opaque vascular casts) were analyzed by microCT, and a first 3D model was reconstructed. Bones were then decalcified and reanalyzed by microCT; a second model (comprising only the vessels) was obtained and overimposed on the former, thus providing a clear visualization of vessel trajectories in the invaded metaphysic allowing quantitative evaluation of the vascular volume and vessel diameter. Histological analysis of the marrow was possible on the decalcified specimens. Walker 256/B cells induced a marked osteolysis with cortical perforations. The metaphysis of invaded bones became progressively hypervascular. New vessels replaced the major central medullar artery coming from the diaphyseal shaft. They sprouted from the periosteum and extended into the metastatic area. The newly formed vessels were irregular in diameter, tortuous with a disorganized architecture. A quantitative analysis of vascular volume indicated that neoangiogenesis increased with the development of the tumor with the appearance of vessels with a larger diameter.

**Conclusion:**

This new method evidenced the tumor angiogenesis in 3D at different development times of the metastasis growth. Bone and the vascular bed can be identified by a double reconstruction and allowed a quantitative evaluation of angiogenesis upon time.

## Introduction

Most cancers (prostate, breast, lung…) can metastasize to the skeleton. The primary tumor cannot exceed a certain size (few mm^3^) without being supplied by new blood vessels [1]. Tumor angiogenesis is a necessary proliferation of a network of blood vessels that penetrates into cancerous tissues, supplies nutrients and oxygen and removes waste products [2], [3], [4]. An undesirable consequence is that neovascularization favors cancer cells metastasis; metastatic areas also develop hypervascularization. When localized in the bone marrow, tumor cells release growth factors and cytokines that can modify the microenvironment and the bone remodeling: parathyroid hormone-related protein (PTHrP), transforming growth factor beta (TGFβ) colony stimulating factor (CSF-1), granulocyte-monocyte CSF (GM-CSF), and chemokines. Other growth factors and cytokines found in the microenvironment include TGFβ, platelet-derived growth factor (PDGF), basic fibroblast growth factor (bFGF), interleukins 6 and 8 (IL-6, IL-8) [5], [6]. Most types of human cancer cells also express vascular endothelial growth factor (VEGF), often at elevated levels. Hypoxia, being recognized as a characteristic in solid tumors, is an important inducer of VEGF [7]. Bone metastases are often hypervascularized: in some bone surgeries (e.g. surgical decompression in hypervascular vertebral metastases), embolization with micro beads is required to avoid intra-operative blood loss [8], [9]. In addition, anti-angiogenic drugs have been developed to limit the growth of tumors [10].

The bone matrix is a favorable microenvironment, rich in sequestered growth factors such as bone morphogenetic proteins (BMPs), insulin-like growth factors (IGF-1), and TGFβ. Degradation of bone matrix by osteoclasts releases the entrapped growth factors that, in turn, promote tumor cell proliferation [11], [12], [13], [14]. The vasculature is particular in the bone marrow; it consists of sinusoidal capillaries with a larger diameter than capillaries found in other tissues [15]. Blood flow is reduced allowing an easy adhesion of young blood cells at the vascular surface to favor entering the blood stream [16]. The sinusoidal capillaries have discontinuous walls made of endothelial cells with no tight junctions. Thus, the structure of the marrow sinusoids and the sluggish blood flow make an advantageous route for tumor cells to invade the bone marrow [17], [18].

The aim of this study was to characterize in 3D, the vascular network in bone metastases in the rat by using microcomputed tomography (microCT) at different stages of evolution of the tumor. Injection of a radio-opaque vascular compound was used at physiological pressure to study distribution, density and shape of the blood vessels distributed in osteolytic metastases caused by injection of Walker 256/B cells in the rat. Because the vascular injection compounds have the same (or higher radio-opacity) than bone, a special technique was developed to allow a clear identification of the injected vessels and a quantification in 3D in the metastatic areas.

## Materials and Methods

### Walker 256/B cell line culture

Walker 256/B, a malignant mammary carcinoma cell line capable of inducing bone metastases was used. Cells were kindly provided by Prof. R. Rizzoli (Rehabilitation and Geriatrics, Geneva University Hospitals, Switzerland). They were grown in Dulbecco modified Eagle's medium (DMEM, Eurobio, Courtaboeuf, France), supplemented with 10% of heat-inactivated fetal calf serum (Eurobio), 100 UI/ml penicillin/streptomycin (Eurobio), 1% of nonessential amino acids (Eurobio) and 1 mM of sodium pyruvate (Eurobio). Cells were cultured in a humidified incubator, with a 5% CO_2_ atmosphere and at a temperature of 37°C. To obtain cells with bone trophicity, 10^7^ Walker 256/B cells were serially passaged intraperitoneally at 7-day intervals in Sprague-Dawley rats to obtain malignant ascites. After 6 or 7 days, ascitic fluids were collected, and cells in suspension were usable to induce bone metastases [19].

### Animals

Twelve Sprague-Dawley (Charles River, L'Arbresle, France), three to four months old and weighing between 480 g and 520 g were used in this experiment. Animals were maintained under local vivarium conditions. Briefly, rats were bred and given standard laboratory food (UAR, Villemoison-sur-Orge, France) and water *ad libitum*. The Animal Care and Use board of University of Angers approved all procedures used in this study (authorization # 49028).

Sprague-Dawley rats were anesthetized by a xylazine/ketamine mixture. The hind legs were shaved and an incision was done on the outer side along the femur axis. After moving apart the muscles, a hole was drilled at the mid femur as previously described to produce a localized metastasis [20]. This technique is favored because animals develop a single bone localization without lung or liver metastases that are observed after arterial injections, leading to a rapid death in about 15/17 days. W256 cells (10^4^ in 10 µl of PBS) were inoculated through the hole into the medullar cavity of the left femurs.

Saline was injected on the right femur prepared in a similar way. The holes were filled by Horsley wax in order to maintain cells on the site of injection. The wound was cleaned with povidone iodine to prevent infections, and finally it was closed with clamps. After surgery, rats were randomized in three groups of four animals:

- Group 1, euthanized and perfused with Microfil®, 12 days postsurgery (D12)
- Group 2, euthanized and perfused with Microfil®, 19 days postsurgery (D19)
- Group 3 euthanized and perfused with Microfil®, 26 days post surgery (D26).

### Microfil® infusion

Microfil MV-122 (Flow Tech, Carver, MA) is a silicon rubber compound developed to produce blood vessels casts. A freshly prepared solution of the compound (containing a yellow dye) was prepared and used immediately, according to the manufacturer recommendations. It consisted of 42% of MV-122, 53% of the diluent solution and 5% of a curing agent.

Rats were euthanized with an excess of xylazine and ketamine. The abdominal cavity was opened under a surgical microscope. The abdominal aorta was clamped under the renal arteries in order to inject only the lower half of the body. Cannulation of the abdominal aorta was done with a polyethylene catheter (0.5 mm, internal diameter, Folioplast, Sarcelles, France) and dripped with a heparinized serum (95% physiological serum, 2.5% xylocaine, and 2.5% heparin). Thereafter 10 ml of the accelerated Microfil® mixture were progressively infused with a manual syringe. Adequate filling of the arteries was assessed when the vessels on the gut and abdominal wall appeared filled with the yellow silicone rubber. Previous studies have found that this corresponded to a physiological pressure of 100–120 mm Hg.

The silicone rubber was left to polymerize at room temperature, then animals were fixed *in toto* for one week in formalin. In each animal, both femurs were carefully dissected. When a metastasis has extended in the soft tissues, special attention was paid to leave a large amount of flesh around the bone.

### X-Ray microcomputed tomography (microCT)

MicroCT analyses were performed using a Skyscan 1172 X-ray computed microtomograph (Skyscan, Kontich, Belgium) equipped with an X-ray tube working at 80 kV/100 µA.

In a first time, femurs were analyzed undecalcified: they were placed in an Eppendorf tube filled with water to prevent desiccation. The tube was fixed on a brass stub with plasticine and analyzed at a resolution of 8.29 microns per pixel. The rotation step was fixed at 0.25° and exposure was done with a 0.5 mm aluminum filter. For each sample, a stack of ∼1500 2D-sections was obtained, ranging from the femoral condyle to the injection hole. Femurs were then decalcified during four days in a mixture of formic acid (4%) and 10% formalin. This decalcifying fluid is recommended since it does not soften the bone matrix and does not provoke collagen swelling nor distort the samples [21]. Decalcified femurs were rinsed in tap water to remove acid remnants, and kept in 10% formalin until re-analysis in the same conditions by microCT. A similar stack of 2D-sections (ranging from the condyles to the injection hole), was obtained and contained only the vascular system. During this step, the decalcified samples were placed in a similar Eppendorf tube filled with formalin.

### Construction of 3D models and morphometry

**Figure 1:**
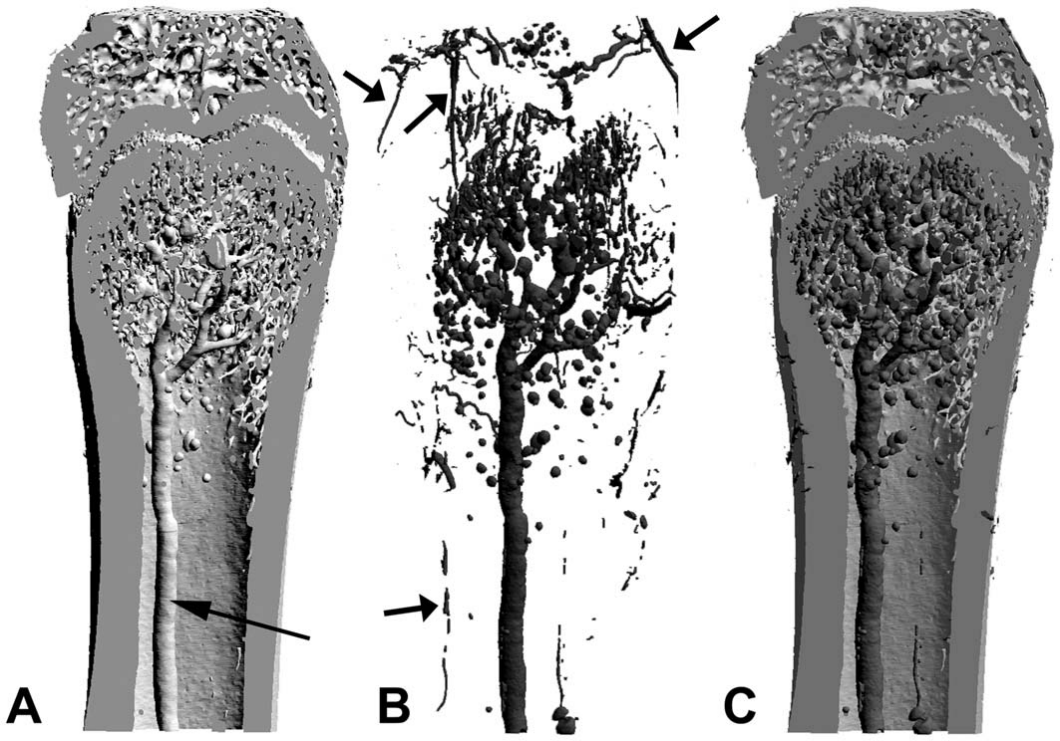
Principle of over imposition of two 3D models. A) 3D model obtained on the undecalcified bone; bone and vascular bed are identified after a single threshold. The arrow points on the central medullar artery. B) 3D model of the vascular bed obtained after decalcification of the same femur. Periosteal arteries surrounding the bone shaft and epiphysis are evidenced (→). C) Overimposition of the two 3D models. The vascular bed appears with a different pseudo-color than bone.

The ANT Software (Skyscan, release 2.2) was used for building 3D models. Ant is a surface-rendering program: 3D models are constructed from the stacks of 2D images after thresholding of the relevant objects from the background noise. For each femur of each animal, the 3D model of the undecalcified bone (containing the calcified bone matrix and the vascular cast) was first prepared (Fig. 1A). Another 3D model, comprising the vessels alone, was obtained on the decalcified bones (the decalcified matrix being radiolucent) (Fig. 1B). Both models were aligned and mixed in the ANT software by overimposing the larger vessels visible in both models. Pseudocolors were assigned to the two models to provide a clear-cut difference between bone and the vascular cast (Fig. 1C). A cutting plane was used to trim the models and highlight the areas of interest. The relative volume occupied by the vessels and the frequency distribution of their diameter were measured in 3D with the CtAn software (Skyscan) after determination of a volume of interest (VOI) starting under the growth plate and extending 13 mm below in the diaphysis. For each model, the volume of interest (VOI) was designed by drawing interactively polygons on the 2D sections. Only on a few number of polygons need to be drawn (e.g. starting, several at the middle, and on final sections) since a routine facility calculated all the intermediary masks by interpolation. The VOI comprised the cortical bone and the marrow cavity. Vessels were identified after thresholding and the vascular volume in the VOI was determined by the software. For determining the diameter of the vessels, the sphere algorithm (implemented in the CtAn software) and routinely used to measure the mean bone trabecular thickness was used [22]. Briefly, the maximal diameter of non-overlapping spheres filling the vessels was computed and the frequency distribution of their diameter was obtained. Data were pooled in each group for all observational times and were transferred to TableCurve (release 5.01, Systat Software) to obtain smoothened curves. Statistical analysis was performed using Systat® statistical software release 11.0 (Systat Software, San Jose, CA). Differences among groups were searched with the Kruskal-Wallis One-Way Analysis of Variance and differences between groups by Mann-Whitney's U test when the ANOVA revealed a significant difference. Differences were considered as significant when p<0.05. Results are expressed as mean ± one standard deviation.

### Histology

Decalcified femurs were dehydrated in ethanol, cleared in xylene and embedded in paraffin and longitudinally sectioned (7 µm of thickness). Sections were stained with hematoxylin-phloxin, rinsed in distilled water and mounted. Sections were evaluated using light microscopy on a LEITZ DMR microscope (Leica Microsystems, Rueil-Malmaison, France).

## Results

### MicroCT analysis

**Figure 2:**
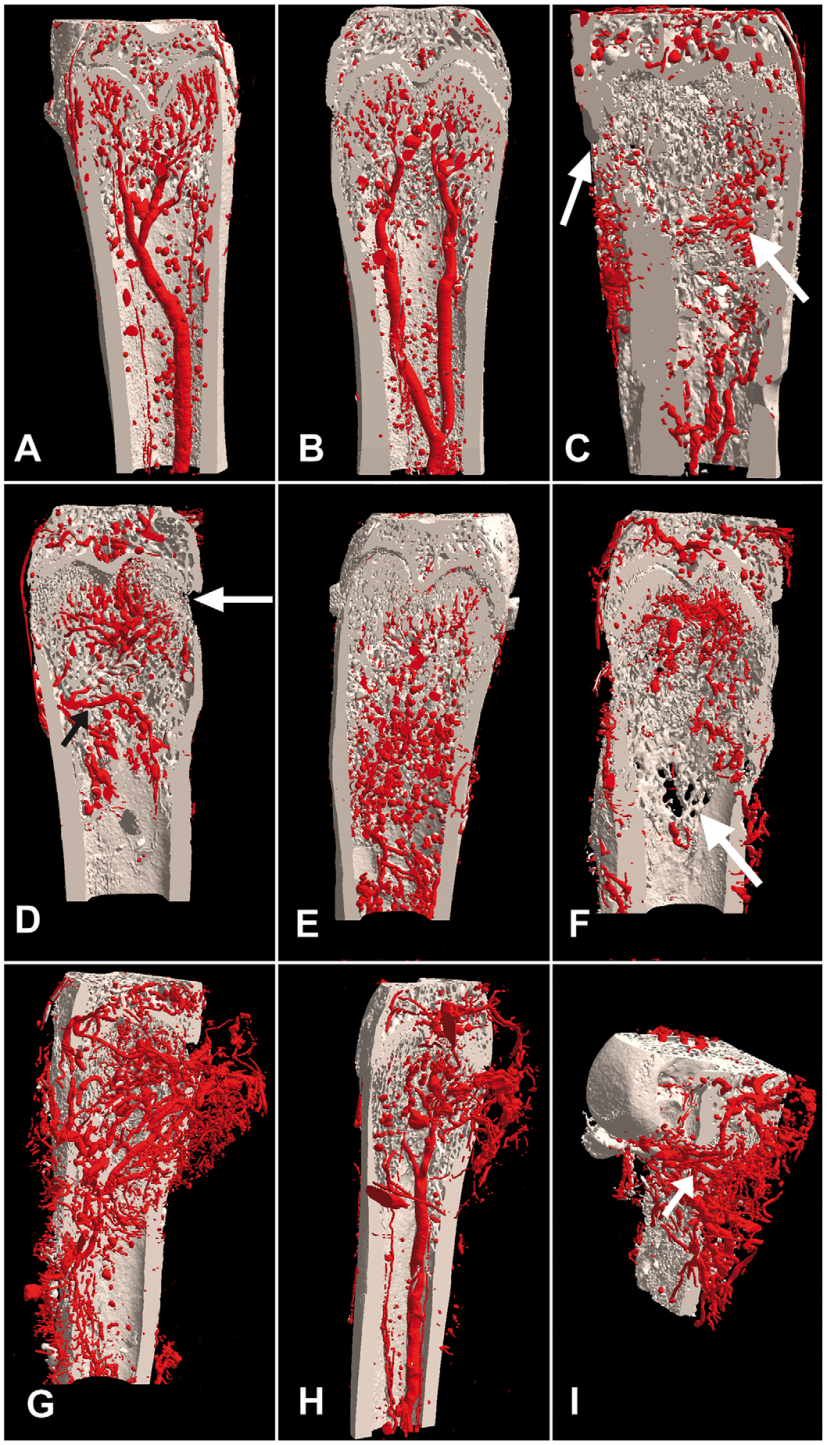
3D models of the vascularization in the rat femur after Microfil® injection. (A) In control femurs, the 3D vascular system is composed of one diaphyseal artery and its branches extending at metaphysis. (B) Two arteries running in parallel in the same femoral shaft were observed in some animals. (C) At day 12 days post-inoculation with W256/B cells, small, winding and tortuous arteries have sprouted from the periosteum and replaced the main nutrient artery in the diaphyseal shaft (↖). Note the thinning of cortices (↑). (D) At D19, irregular blood vessels come from the periosteum (↗). Osteolysis in the primary spongiosa and cortical perforations (↑) are evidenced. (E) At D26, arteries with a chaotic microarchitecture have invaded the whole metaphysis. (F) Osteolysis with cortical perforation and trabecular destruction becomes obvious (↑). (G) Comparison between the metastatic femur, which became hypervascular and the normal controlateral femur (H) evidences the considerable difference in the vascular beds. (I) Several arteries coming from the periosteal surface appeared the main suppliers of blood in the metaphysis (↑).

The right femurs, without injection of malignant cells, were used as control; the 3D vascular network consisted of a nutrient artery running longitudinally in the central marrow cavity of the diaphyseal shaft (Fig. 2). This artery divided into two or three branches before entering in the metaphysis. In the metaphysis, these branches divided in turn into numerous sinusoid capillaries that extended through the trabeculae of the cancellous bone (Fig. 2A). Occasionally, two arteries were observed in the same femur in two animals (Fig. 2B). Arteries and capillaries had a regular shape, the same thickness, and a well-organized orientation. Femurs had a smooth periosteal surface and the thickness of the cortex was regular. Bone trabeculae had a regular microarchitecture in both the primary and secondary spongiosa and the growth plate was clearly evidenced as a radiolucent stripe between the epiphysis and the metaphysis.

In group 1, (12 days after inoculation of W256/B cells), the nutrient artery and its branches have disappeared and were replaced by small arteries. They appeared winding and tortuous with an irregular diameter, and extending in all directions (Fig. 2C). The vasculature was simultaneously developed on the periosteal surface and in the surrounding soft tissues. At this stage, there was no noticeable change in bone microarchitecture.

In group 2, (19 days after inoculation of W256/B cells), bone vascularization had dramatically increased; vessels were more numerous in the diaphyseal area (Fig. 2D). Blood vessels were tortuous and some were considerably increased in diameter. In the absence of the nutrient diaphyseal artery, the blood supply came from a large metaphyseal artery coming from the periosteum. Perforations of the cortical bone were observed and osteolysis developed in the primary spongiosa. In one animal, metaplastic bone developed at the periosteal surface with a sunburst appearance.

In group 3, (26 days after inoculation of W256/B cells), blood vessels had invaded the whole diaphysis (Fig. 2E). A marked osteolysis occurred in the primary and secondary spongiosa. Cortical perforations were numerous and easily observable on the 3D models (Fig. 2F). Femurs appeared hypervascular (Fig. 2G and 2H). Vessels were tortuous and an artery (with a larger diameter than the diaphyseal artery seen in the controlateral femur), was vascularizing the metastatic site. Several arteries, coming from the periosteal surface, were increased in diameter inside the metaphysis and constituted the main supply of blood in the metaphysis (Fig. 2I). The 3D organization of the blood vessels was irregular or even chaotic. Microfil® passage into the medullary sinusoid capillary formed round microbeads. Neo-vascularization extended over the periosteal surface and developed into the surrounding soft tissues. A marked osteolysis occurred in the primary and secondary spongiosa but could not be quantified by histomorphometry due to the impossibility to threshold bone from the vascular cast. Cortical perforations were numerous and easily observable on the 3D models (Fig. 2F). Periosteal surfaces contained a considerable number of arteries. Metaplastic trabecular bone was more frequently observed at the periosteal surface.

**Figure 3:**
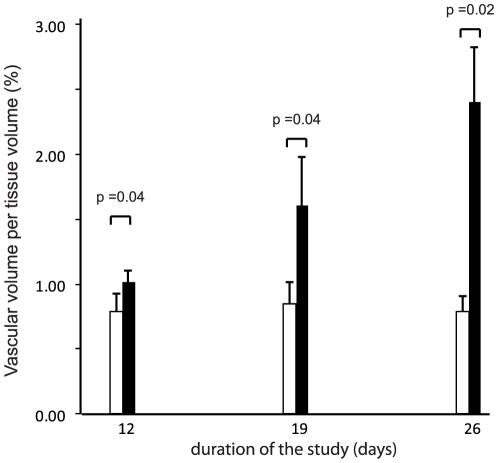
Time evolution of vascular volume in the control and metastatic femurs.

**Figure 4:**
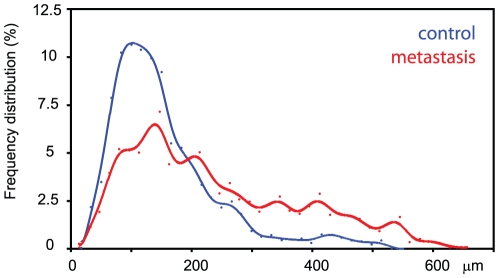
Frequency distribution of the vascular diameter on the control side (in blue) and the metastatic side (in red). In controls, the arteries appeared to be centered on a peak at 100–120 µm whatever the time of analysis. In the metastatic side, larger arteries (up to 600 µm in diameter) were observed.

The vascular volume per tissue volume was significantly different from the control bones as early as 12 days post injection (p<0.04). On the control bones, the vascular volume did not change upon time; on the contrary, it considerably increased on the femur with the metastasis (Fig. 3). The frequency distribution of vascular vessels appears on figure 4. On the control side, a peak centered at 100–120 µm was observed at the three times of analysis and corresponded to at least ∼11% of the vessel diameters. Larger values were observed at a low frequency. On the other hand, the frequency distribution curves obtained on the invaded sides exhibited a marked heterogeneity. The highest peak appeared at 120–150 µm but larger vessels were yet identified; some of them appeared considerably enlarged, reaching 600 µm in diameter.

### Histology

**Figure 5:**
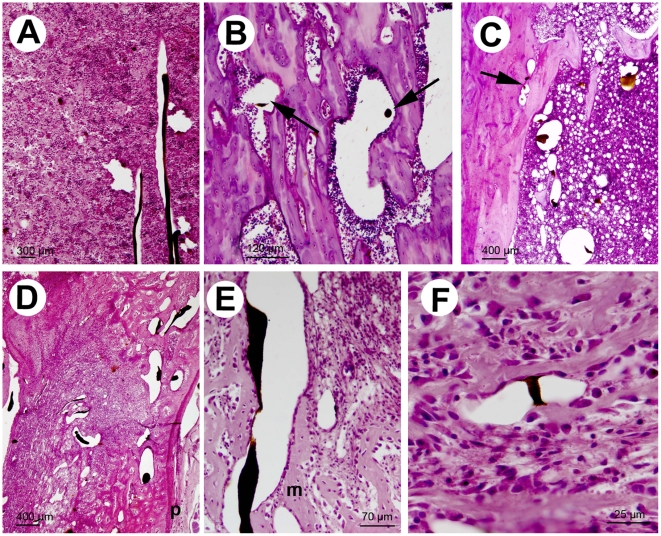
Histological aspect of bones after Microfil® injection and decalcification, Microfil® appears shrunk with a deep gray-black tint. Hematoxylin-phloxin staining. A) Control femur showing two medullar arteries running in parallel cf Fig 2B. B) Control femur showing the silicone rubber in a dilated vascular sinus (right arrow) and in a vascular canal in the cortice (left arrow). C) Metastatic femur at 12 days with enlarged vascular channels in the area of tumors cells, arrow points to an intracortical vessel. D) Metastatic femur at 26 days (see Fig 2G) with cortical perforation, large vessels coming from the periosteum (p) and extending in the tumor area having a fibrous stroma and foci of metaplastic bone. E) A large vessel running between metaplastic trabeculae (m) and tumor cells. F) Tumor cells with a spindle-shape or enlarged cytoplasm, thin metaplastic trabeculae and a vessel containing Microfil®.

In healthy femurs, trabeculae had a regular microarchitecture: trabeculae were thin and parallel in the primary spongiosa; they were larger in the secondary spongiosa (made of plate and pillars). The bone cortices had a regular thickness that decreased from the mid femoral shaft to the area of the growth plate (Ranvier's metaphyseal groove). The bone marrow was composed by a dense population of cells of the hematopoietic lineage intercalated with adipocytes. Microfil® was not dissolved by solvents used in the histotechnological processing. It remained visible either as pseudo-circular or elongated profiles, depending on the position of blood vessels in relation to the cutting plane of microtome knife (Fig. 5). Some parts were closely attached to the trabeculae. However, Microfil® appeared torned, shrunken and folded, and therefore did not fit with the spatial form of the blood vessels whose distended wall remained visible. Due to the considerable shrinkage induced by clearing in xylene during the histotechnological step, it was not possible to correlate precisely the 2D and 3D data on the vasculature. In metastatic femurs, the bone marrow was invaded by tumor cells having a spindle-shape. Microfil® was visible in the metastasis, the soft tissues invaded by tumor cells or between metaplastic trabeculae formed on the periosteal surface.

## Discussion

Tumor angiogenesis is important for tumor growth and metastatic dissemination. There are differences between tumor and normal blood vessels, the later having abnormal architecture and an irregular blood flow [23]. In the golden hamster, a study on tumor grown in dorsal skinfold chambers revealed a sluggish flow of the blood circulation and tortuous microvessels with a chaotic architecture [24]. In another study on neovascularization of VX2 liver tumors in the rabbit, the authors reported that blood vessels had a tortuous and meandering form in the tumor area compared to those of non-invaded areas [25]. Different methods have been used to characterize the architecture of tumor vasculature in animal studies: histology or X-ray microangiography are the most common [26], [27]. However, histology is based on 2D sections and is not representative of the vessel trajectories throughout the entire sample. X-ray microangiography lacks volumetric analysis although the method can identify small capillaries by using highly radio-opaque materials such as barium sulfate [28], [29]. Therefore, a 3D characterization of tumor vasculature is necessary because it provides valuable anatomical data. Barium sulfate, iodine and Microfil® are the most frequently used contrast media to visualize vascularization in 2D and 3D [30], [31]. Their field of use is large and includes as well as heart with coronary angiography, lung cancer [32], kidney diseases [33] or neoplastic disorders. For example, microangioarchitecture of VX2 tumors in the ears of the rabbit was evaluated after injecting 10% barium sulfate in the auricular artery; microCT improved the detection of fine blood vessels compared to conventional radiography [34]. Although, barium sulfate and iodine give excellent contrast with soft tissue, they are not suitable when larger blood vessels are present since they provoke reconstruction artifacts on the microCT images. Here, Microfil® appeared to have a suitable radio-opacity and did not induce reconstruction artifacts on the larger bone arteries. Bone matrix is composed of two phases: collagenous (radiolucent) and mineral (radio-opaque); the later prevents a clear identification of vascularization in 3D excepted in the larger central arteries of the bone marrow. Therefore, decalcification was used here to remove the mineral phase; it enabled a good visualization of the vascular network without shrinkage. The need to overimpose the two 3D models (bone + vascularization and vascularization alone) was facilitated by aligning the larger vessels in 3D and the capacity of the ANT software to allow a precise overimposition of the two models at the voxel level. Decalcification is not compatible with the use of liquid contrast media, such as barium sulfate, because blood vessels are not rigid and collapse; Microfil® is a silicone polymerisable rubber that produces semi-rigid vascular casts that do not distort. Injection in the abdominal aorta was performed with a large volume of Microfil® because visual or radiographic controls were not possible to see if the casting mixture had reached the small blood vessels inside the bone marrow. A study characterizing the microvascular bed in the bone marrow was done in adult dogs after perfusion of gelatin containing india ink. It revealed a closed capillary system with a thin and continuous endothelium lying on a basal lamina [35]. These capillary sinuses have been the subject of a considerable interest since they appear to be formed on flat (endothelial) cells on the marrow side and by lining cells (flattened osteoblasts) on the bone surfaces [36], [37]. The physiological role of these anatomical structures is not fully understood, they are necessary for bone remodeling (the “canopy” theory) but the blood flow inside their lumen remains to be fully explored. The globular aspects observed on some microCT images may represent a partial filling of these systems. A recent study demonstrated a simultaneous visualization of bone and normal bone vasculature in mice. Assays were performed by synchrotron and vascular casting corrosion, or vascular contrast perfusion, were used [38]. For vascular casting corrosion less than 10 mL of PU4ii, a polyurethane-based casting resin, were used and injected into the left heart ventricle, while 5 mL of barium sulfate were used for the vascular perfusion. In both protocols, the vascular system was previously pre-fixed with a perfusion of paraformaldehyde [38]. However, these studies have only explored the medullar and cortical vascularization of normal animals. Recently, Microfil® was used to evaluate the vascularization associated with activation of Bmp signaling in mouse osteoblasts. However, the bones were decalcified by formic acid before microCT analysis of the vascular bed [39].

Metastatic bone lesions obtained by W256\B cells form a large osteolytic band in the primary spongiosa and cortical perforations are frequent. Osteolysis is visible on X-ray images, as a radiolucent area just below the growth plate [19], [20], [40]. On histological sections, tumor nodules are also evidenced in the secondary spongiosa [20]. Angiogenesis is essential for bone formation, bone remodeling, and bone healing [41]. In bone remodeling, the newly formed blood vessels can serve as pathways for osteoclast and osteoblast precursors to remodeling sites [42].

In the present study, we found a vascular architecture in healthy femora consisting in a major nutrient artery with metaphyseal branches, and capillaries, although two arteries were sometimes evidenced. These observations are in accordance with a previous report [17]. Invaded femurs were hyper vascularized as shown by quantitative evaluation of the vascular bed; the main feature was the disappearance of the nutrient artery and the development of a collateral network coming from the periosteum. The neo-vascularization started in the group 1 with abnormal blood vessels sprouting in diaphyseal area. At that time, there was no noticeable difference between healthy and metastatic femurs concerning bone microarchitecture. In the group 2, more vessels were observed and osteolysis could be observed at this stage. A considerable increase in the vascular bed occurred in the group 3. Bloods vessels invaded the whole metaphysis, extended over the periosteal surfaces and in the surrounding soft tissues. In all groups, tumor blood vessels were tortuous and presented a disorganized microarchitecture. Analysis of the frequency distribution of the vascular diameter confirmed the appearance of enlarged vessels at the tumor site. The vessels were found to reach 600 µm in diameter while most of the arteries were 100–120 µm on the control side. We have previously found that the sphere algorithm used in the CtAn software to compute the mean diameter of bone trabeculae is more interesting in a number of cases when the distribution of data is of importance (e.g. the distribution of trabecular thickness in osteoporosis or the macro/microporosity in biomaterials) [43], [44]. Similar observations have been repeatedly found in the literature. In a study on gastric carcinoma comparing differentiated and undifferentiated carcinomas, the vascular beds were perfused with Microfil® and vascular irregularities like winding, meandering and irregular vascular diameter were found mostly in undifferentiated carcinomas [45]. In addition, blood vessels are known to be anatomically defective, with irregular shapes, dilatations, and endothelial cells forming a discontinuous wall [46]. Histological observations did not allow a good visualization of the Microfil® localization. It appeared torn and folded on all sections due to the shrinkage of the silicone induced by the histotechnological steps.

In this study, the 3D microarchitecture of the vasculature was characterized in the rat femur at different times of development of an osteolytic metastasis. W256/B cells induced a marked increase in the vascular bed at the metaphysis. Newly formed blood vessels were irregular and enlarged in diameter, tortuous with a disorganized architecture and developed from the periosteum.

## References

[1] Folkman J (1995). Angiogenesis in cancer, vascular, rheumatoid and other disease.. Nature Med.

[2] Jain RK (2003). Molecular regulation of vessel maturation.. Nature Med.

[3] Liao D, Johnson RS (2007). Hypoxia: A key regulator of angiogenesis in cancer.. Cancer Metast Rev.

[4] Kerbel RS (2008). Tumor angiogenesis.. N Engl J Med.

[5] Chantrain CF, Feron O, Marbaix E, Declerck YA (2008). Bone marrow microenvironment and tumor progression.. Cancer Microenviron.

[6] Lorusso G, Rüegg C (2008). The tumor microenvironment and its contribution to tumor evolution toward metastasis.. Histochem Cell Biol.

[7] Semenza GL (2003). Angiogenesis in ischemic and neoplastic disorders.. Annu Rev Med.

[8] Guzman R, Dubach-Schwizer S, Heini P, Lovblad KO, Kalbermatten D (2005). Preoperative transarterial embolization of vertebral metastases.. Eur Spine J.

[9] Schirmer CM, Malek AM, Kwan ES, Hoit DA, Weller SJ (2006). Preoperative embolization of hypervascular spinal metastases using percutaneous direct injection with N-butyl cyanoacrylate: Technical case report.. Neurosurgery.

[10] Jain RK (2005). Normalization of tumor vasculature: An emerging concept in antiangiogenic therapy.. Science.

[11] Joyce JA, Pollard JW (2009). Microenvironmental regulation of metastasis.. Nat Rev Cancer.

[12] Guise TA, Chirgwin JM (2003). Transforming growth factor-beta in osteolytic breast cancer bone metastases.. Clin Orthop Relat Res.

[13] Guise TA, Mundy GR (1998). Cancer and bone.. Endocr Rev.

[14] Mundy GR (2002). Metastasis to bone: causes, consequences and therapeutic opportunities.. Nat Rev Cancer.

[15] Pugsley MK, Tabrizchi R (2000). The vascular system. An overview of structure and function.. J Pharmacol Toxicol Methods.

[16] Mastro AM, Gay CV, Welch DR (2003). The skeleton as a unique environment for breast cancer cells.. Clin Exp Metast.

[17] Mazo IB, Von Andrian UH (1999). Adhesion and homing of blood-borne cells in bone marrow microvessels.. J Leukocyte Biol.

[18] Bussard KM, Gay CV, Mastro AM (2008). The bone microenvironment in metastasis; what is special about bone?. Cancer Metastasis Rev.

[19] Blouin S, Moreau MF, Baslé MF, Chappard D (2006). Relations between radiograph texture analysis and microcomputed tomography in two rat models of bone metastases.. Cells Tissues Organs.

[20] Blouin S, Baslé MF, Chappard D (2005). Rat models of bone metastases.. Clin Exp Metastasis.

[21] Ganter P, Jollès G (1969). Histochimie normale et pathologique..

[22] Hildebrand T, Ruegsegger P (1997). A new method for the model-independent assessment of thickness in three-dimensional images.. J Microsc.

[23] Chen Q, Wang WC, Evans SS (2003). Tumor microvasculature as a barrier to antitumor immunity.. Cancer Immunology, Immunotherapy.

[24] Strieth S, Strelczyk D, Eichhorn ME, Dellian M, Luedemann S (2008). Static magnetic fields induce blood flow decrease and platelet adherence in tumor microvessels.. Cancer Biol Ther.

[25] Sugimoto K, Moriyasu F, Kamiyama N, Metoki R, Iijima H (2007). Parametric imaging of contrast ultrasound for the evaluation of neovascularization in liver tumors.. Hepatol Res.

[26] Yamashita T (2001). Evaluation of the microangioarchitecture of tumors by use of monochromatic x-rays.. Invest Radiol.

[27] Silvestre JS, Tamarat R, Ebrahimian TG, Le-Roux A, Clergue M (2003). Vascular endothelial growth factor-B promotes in vivo angiogenesis.. Circulation Res.

[28] Baron-Menguy C, Bocquet A, Guihot AL, Chappard D, Amiot MJ (2007). Effects of red wine polyphenols on postischemic neovascularization model in rats: low doses are proangiogenic, high doses anti-angiogenic.. Faseb J.

[29] Duvall CL, Taylor WR, Weiss D, Guldberg RE (2004). Quantitative microcomputed tomography analysis of collateral vessel development after ischemic injury.. Am J Physiol Heart Circ Physiol.

[30] Wietholt C, Li J, Aydogan B, Archer SL, Rajh T (2008). Comparison of CT contrast blood pool agents for in-vivo 3D angiography using microCT.. IEEE Nucl Sci Symp Conf Rec art.N°.

[31] Marxen M, Thornton MM, Chiarot CB, Klement G, Koprivnikar J (2004). MicroCT scanner performance and considerations for vascular specimen imaging.. Med Phys.

[32] Savai R, Langheinrich AC, Schermuly RT, Pullamsetti SS, Dumitrascu R (2009). Evaluation of angiogenesis using micro-computed tomography in a xenograft mouse model of lung cancer.. Neoplasia.

[33] Nieman K, Oudkerk M, Rensing BJ, Van Ooijen P, Munne A (2001). Coronary angiography with multi-slice computed tomography.. Lancet.

[34] Maehara N (2003). Experimental microcomputed tomography study of the 3D microangioarchitecture of tumors.. Eur Radiol.

[35] Miller SC, Jee WSS (1980). The microvascular bed of fatty bone marrow in the adult beagle.. Metab Bone Dis Rel Res.

[36] Hauge EM, Qvesel D, Eriksen EF, Mosekilde L, Melsen F (2001). Cancellous bone remodeling occurs in specialized compartments lined by cells expressing osteoblastic markers.. J Bone Miner Res.

[37] Andersen TL, Sondergaard TE, Skorzynska KE, Dagnaes-Hansen F, Plesner TL (2009). A physical mechanism for coupling bone resorption and formation in adult human bone.. Am J Pathol.

[38] Schneider P, Krucker T, Meyer E, Ulmann-Schuler A, Weber B (2009). Simultaneous 3D visualization and quantification of murine bone and bone vasculature using micro-computed tomography and vascular replica.. Microsc Res Techn.

[39] Zhang F, Qiu T, Wu X, Wan C, Shi W (2009). Sustained BMP signaling in osteoblasts stimulates bone formation by promoting angiogenesis and osteoblast differentiation.. J Bone Miner Res.

[40] Badraoui R, Blouin S, Moreau MF, Gallois Y, Rebai T (2009). Effect of alpha tocopherol acetate in Walker 256/B cells-induced oxidative damage in a rat model of breast cancer skeletal metastases.. Chem Biol Interact.

[41] Brandi ML, Collin-Osdoby P (2006). Vascular biology and the skeleton.. J Bone Miner Res.

[42] Eriksen EF, Eghbali-Fatourechi GZ, Khosla S (2007). Remodeling and vascular spaces in bone.. J Bone Miner Res.

[43] Chappard D, Retailleau-Gaborit N, Legrand E, Baslé MF, Audran M (2005). Comparison insight bone measurements by histomorphometry and microCT.. J Bone Miner Res.

[44] Filmon R, Retailleau-Gaborit N, Brossard G, Grizon-Pascaretti F, Baslé MF (2009). Preparation of β-TCP granular material by polyurethane foam technology.. Image Anal Stereol.

[45] Adachi Y, Mori M, Enjoji M, Sugimachi K (1993). Microvascular architecture of early gastric carcinoma: Microvascular- histopathologic correlates.. Cancer.

[46] Iyer AK, Khaled G, Fang J, Maeda H (2006). Exploiting the enhanced permeability and retention effect for tumor targeting.. Drug Disc Today.

